# Regulation of Effector Delivery by Type III Secretion Chaperone Proteins in *Erwinia amylovora*

**DOI:** 10.3389/fmicb.2018.00146

**Published:** 2018-02-08

**Authors:** Luisa F. Castiblanco, Lindsay R. Triplett, George W. Sundin

**Affiliations:** ^1^Department of Plant, Soil and Microbial Sciences, Center for Microbial Pathogenesis, Michigan State University, East Lansing, MI, United States; ^2^Department of Plant Pathology and Ecology, The Connecticut Agricultural Experiment Station, New Haven, CT, United States

**Keywords:** type III secretion, type III chaperones, class IB type III chaperone proteins, effector translocation, *Erwinia amylovora*

## Abstract

Type III secretion (TTS) chaperones are critical for the delivery of many effector proteins from Gram-negative bacterial pathogens into host cells, functioning in the stabilization and hierarchical delivery of the effectors to the type III secretion system (TTSS). The plant pathogen *Erwinia amylovora* secretes at least four TTS effector proteins: DspE, Eop1, Eop3, and Eop4. DspE specifically interacts with the TTS chaperone protein DspF, which stabilizes the effector protein in the cytoplasm and promotes its efficient translocation through the TTSS. However, the role of *E. amylovora* chaperones in regulating the delivery of other secreted effectors is unknown. In this study, we identified functional interactions between the effector proteins DspE, Eop1, and Eop3 with the TTS chaperones DspF, Esc1 and Esc3 in yeast. Using site-directed mutagenesis, secretion, and translocation assays, we demonstrated that the three TTS chaperones have additive roles for the secretion and translocation of DspE into plant cells whereas DspF negatively affects the translocation of Eop1 and Eop3. Collectively, these results indicate that TTS chaperone proteins exhibit a cooperative behavior to orchestrate the effector secretion and translocation dynamics in *E. amylovora*.

## Introduction

The delivery of effector proteins via the type III secretion system (TTSS) is a critical step for pathogenesis of many Gram-negative bacteria (Büttner and He, 2009; Raymond et al., 2013; Galán et al., 2014). The secretion signal is typically localized in the N-terminal domain of the effector or within the 5′ region of the effector mRNA (Miao and Miller, 2000; Mudgett et al., 2000; Blaylock et al., 2008). Loosely conserved sequence or structural patterns in this region can be used in computational approaches for *in silico* prediction of type III secreted substrates (Guttman et al., 2002; Petnicki-Ocwieja et al., 2002). The translocation efficiency of many effector proteins also depends on a physical association with cytoplasmic type III secretion (TTS) chaperone proteins (Luo et al., 2001; Parsot et al., 2003; Matsumoto and Young, 2009; Triplett et al., 2009). TTS chaperones are typically low molecular weight acidic proteins that remain within the bacterial cytoplasm and form dimeric or hexameric structures that bind to their target effectors (Thomas et al., 2012; Tsai et al., 2015). TTS chaperones are categorized into three groups: class I TTS chaperones bind to effector proteins, class II TTS chaperones bind to type III pore-forming (translocon) proteins, and class III TTS chaperones bind to needle proteins (Cornelis and Gijsegem, 2000; Parsot et al., 2003). Two subclasses are found within class I TTS chaperones: class IA chaperones bind to single effector proteins, and class IB chaperones bind to multiple effectors (reviewed in Thomas et al., 2012). Despite exhibiting low overall amino acid residue similarity, TTS chaperones share a conserved three-dimensional structure and similar modes of interaction with their effector partners (Ghosh, 2004; Cornelis, 2006). These similarities have been used to model the three-dimensional structure of chaperone proteins from plant pathogens, as well as to identify critical residues in the interaction with their cognate effectors (Triplett et al., 2010).

Type III secretion chaperone binding may directly protect effectors from degradation by the Lon or other proteases in permissive conditions for TTS (Losada and Hutcheson, 2005). In addition, TTS chaperones in diverse bacteria interact with ATPases associated with the TTSS. This interaction induces the docking, unfolding and release of the effector protein to the secretion system (Akeda and Galán, 2005; Lorenz and Buttner, 2009; Cooper et al., 2010). The TTS chaperone HpaB from *Xanthomonas campestris* pv. *vesicatoria* establishes a secretion hierarchy that allows the secretion of TTSS components prior to that of effector proteins (Lorenz et al., 2008). TTS chaperones may also interact with non-secreted proteins, such as transcription factors, in order to upregulate the expression of effector genes and facilitate the global regulation of the TTS (Darwin and Miller, 2001).

*Erwinia amylovora*, the causal agent of fire blight disease of rosaceous plants including apple and pear (Malnoy et al., 2012) secretes at least four effector proteins: DspA/E (DspE henceforth), Eop1, AvrRpt2_Ea_/Eop4 (Eop4 henceforth) and Eop3 (Bogdanove et al., 1998; Zhao et al., 2006; Nissinen et al., 2007). Among these, only DspE is required for pathogenicity, multiplication *in planta*, and for disease promotion by the alteration of host defenses, inducing cell death in both host and non-host plants (Gaudriault et al., 1997; Boureau et al., 2006). DspE interacts with the TTS chaperone protein DspF, which stabilizes the effector and prevents its degradation in the cytoplasm, and promotes its efficient translocation through the TTSS (Gaudriault et al., 2002). However, a *dspF* mutant does not lack pathogenic ability, but exhibits reduced aggressiveness and is still able to translocate the N terminal region of DspE (Triplett et al., 2009; Oh et al., 2010), suggesting that other proteins may be involved in the secretion of this effector protein in the absence of or in addition to DspF. The effector protein Eop1, a member of the YopJ family of proteins, is also translocated via the TTSS. Like *dspE*, the *eop1* gene is located adjacent to a TTS chaperone gene, named *orfA* (Oh and Beer, 2005). The *orfA* product interacts not only with Eop1 but also with DspE in yeast (Asselin et al., 2006), suggesting that TTS chaperones in *E. amylovora* may be involved in the translocation of several effectors. The roles of chaperones other than DspF in the regulation of *E. amylovora* effector translocation are unknown.

Understanding the dynamic roles of TTS chaperones during plant pathogenesis is challenging due to the large number of TTS effectors in many model bacterial pathogens. Conversely, the small number of effectors in *E. amylovora* makes it well-suited for understanding the global secretory roles of TTS chaperones in plant pathogens. In this report, we investigated the effect of TTS chaperones on all known effector proteins of *E. amylovora*. We identified novel functional interactions between the effector proteins DspE, Eop1, and Eop3 with their cognate and non-cognate predicted TTS chaperones. We then analyzed the individual and collective effects of these chaperones on secretion, host translocation, and pathogenicity, and demonstrated that TTS chaperones act cooperatively in the regulation of *E. amylovora* effector translocation dynamics.

## Materials and Methods

### Bacterial Strains, Plasmids, Growth Conditions, and Genetic Techniques

The bacterial strains and plasmids used in this study are listed in **Table 1**. Bacteria were grown at 28°C in Luria-Bertani (LB) broth and agar unless otherwise noticed. Media were amended with ampicillin (Amp; 50 mg L^-1^), chloramphenicol (Cm; 10 mg L^-1^), gentamicin (Gm; 10 mg L^-1^) or kanamycin (Km; 25 mg L^-1^) as necessary. PCR, restriction digestions, gene cloning and gel electrophoresis were performed according to standard methods (Sambrook et al., 2001).

**Table 1:** Bacterial strains and plasmids used in this study.

Strain or plasmid	Characteristics^a^	Source or reference
***Escherichia coli* strain**		
DH5α	F-80dlacZ ΔM15 Δ(lacZYA-argF)U169 endA1 recA1 hsdR17 (rk-mk+)deoR thi-1 supE44 gyrA96 relA1 λ-	Invitrogen, Carlsbad, CA, United States
***Erwinia amylovora* strains**		
Ea1189	Wild type	Burse et al., 2004
Ea1189Δ*dspF*	*dspF* deletion mutant, Km^R^	Triplett et al., 2009
Ea1189Δ*esc1*	*esc1* deletion mutant, Cm^R^	This study
Ea1189Δ*esc3*	*esc3* deletion mutant, Cm^R^	This study
Ea1189Δ*dspF/esc1*	*dspF/esc1* deletion mutant, Km^R^ Cm^R^	This study
Ea1189Δ*dspF/esc3*	*dspF/esc3* deletion mutant, Km^R^ Cm^R^	This study
Ea1189Δ*dspF/esc1/esc3*	*dspF/esc1/esc3* deletion mutant, Cm^R^	This study
Plasmids		
pMJH20	pWSK29 containing codons 2 to 406 of CyaA, Amp^R^	Miao and Miller, 2000
pLRT198	pBRR1MCS-5 expressing DspF, Gm^R^	Triplett et al., 2009
pLRT8	pMHJ20 expressing DspE_(1-15)_-CyaA	Triplett et al., 2009
pLRT201	pMHJ20 expressing DspE_(1-737)_-CyaA	Triplett et al., 2009
pLRT177	pMHJ20 expressing Eop1-CyaA	This study
pLRT209	pMHJ20 expressing Eop3-CyaA	This study
pLRT210	pMHJ20 expressing Eop4-CyaA	This study
pGilda	HIS3 LexA BD bait vector, Amp^R^	Clontech, Mountain View, CA, United States
pB42AD	TRP1 B42 AD bait vector, Amp^R^	Clontech
pB42-HA-T	Positive control prey vector, Amp^R^	Clontech
pLexA-53	Positive control bait vector, Amp^R^	Clontech
pLRT192	LexA-DspE_(1-800)_	Triplett et al., 2009
pLRT13	LexA-DspE_(1-150)_	Triplett et al., 2009
pLFC67	LexA-DspE_(738-1838)_	This study
pLRT111	LexA-Eop1	This study
pLRT169	LexA-Eop1_(1-200)_	This study
pLRT231	LexA-Eop1_(135-263)_	This study
pLRT232	LexA-Eop1_(264-402)_	This study
pLRT167	LexA-Eop3	This study
pLRT168	LexA-Eop4	This study
pLRT215	B42-HA-DspF	Triplett et al., 2009
pLRT226	B42-HA-Esc1	This study
pLRT227	B42-HA-Esc3	This study
pKD46	*Red*, β, λ and *exo* recombinases, Amp^R^	Datsenko and Wanner, 2000
pKD3	Mutagenesis cassette template, Cm^R^	Datsenko and Wanner, 2000
pKD4	Mutagenesis cassette template, Km^R^	Datsenko and Wanner, 2000
pCP20	Temperature sensitive replication and induction of FLP synthesis, Amp^R^	Cherepanov and Wackernagel, 1995

*^*a*^Km^*R*^, Cm^*R*^, Gm^*R*^, and Amp^*R*^ indicate resistance to kanamycin, chloramphenicol, gentamicin and ampicillin, respectively.*

### Mutant Construction

Chromosomal mutants were constructed using an adaptation of the λ red recombinase system as previously described (Datsenko and Wanner, 2000; Zhao et al., 2005; Triplett et al., 2009). Briefly, Cm and Km resistance cassettes were amplified from template plasmids pKD3 and pKD4 using primers with 50 bp overhangs, homologous with the gene of interest. PCR products were purified and electroporated into *E. amylovora* wild-type (WT) strain Ea1189 expressing the genes of red, β, λ and exo recombinases from the pKD46 plasmid. Resultant colonies were screened for antibiotic resistance, and gene disruption was verified by PCR and sequencing. In order to create triple and quadruple mutants, pKD46 was cured from Ea1189ΔdspF/esc3 and Ea1189ΔdspF/esc1/esc3 strains by repetitive growth cycles without antibiotic selection and including heat shock at 37°C. Cured strains were transformed with pCP20 in order to resolve antibiotic resistances by the thermo-inducible resolvase encoded in this plasmid. Transformants were tested for Amp, Cm and Km sensitivity prior to initiating the next round of mutagenesis.

### Pathogenicity Assays

Strain pathogenicity was evaluated using immature pear fruit assays as previously described (Zhao et al., 2005; Koczan et al., 2011). Briefly, bacterial suspensions were grown overnight and adjusted to ≈ 1 × 10^4^ CFU mL^-1^ in 0.5x sterile phosphate-buffered saline (PBS). Three microliters of the bacterial suspension were inoculated on previously stab-wounded surface-sterilized immature pears and incubated at 28°C. ImageJ software (National Institutes of Health; Bethesda, MD, United States) was used to quantify lesion area at 4 days post-inoculation (dpi). Pear assays were done in triplicate, and each experiment was repeated at least three times. For evaluation of hypersensitive-like cell death, overnight bacterial suspensions were adjusted to ≈ 1 × 10^7^ CFU mL^-1^ in 0.5x PBS and infiltrated into 8-week old *Nicotiana tabacum* cv. Samsun leaves, using a needleless syringe. Cell collapse was evaluated 24 h post-infiltration (hpi). This assay was done in triplicate and each experiment was repeated at least three times. Statistical analyses were done using a one-way analysis of variance, and mean separation was accomplished using the Tukey–Kramer HDS test using JMP 12 (Cary, NC, United States).

### Yeast Two-Hybrid Assays

*dspE, eop3, eop4*, and *eop1* (full-gene and fragments) were cloned in fusion with the LexA binding domain into the bait vector pGilda (Clontech; Mountain View, CA, United States) using BamHI and XhoI restriction sites. *esc1, esc3*, and *hrpN* were digested with BamHI and EcoRI and cloned into the prey vector pB42AD. Prey and bait constructs were co-transformed into *Saccharomyces cerevisiae* EGY48 (pLacZ) using the Frozen-EZ Yeast Transformation II Kit (Zymo Research Corporation; Irvine, CA, United States). Transformants were selected on minimal synthetic dropout (SD)-galactose/raffinose medium amended with -Ura/-His/-Trp dropout. Positive interaction was screened on SD-galactose medium amended with -Ura/-His/-Trp/-Leu dropout and 5-bromo-4-chloro-3-indolyl-β-galactopyranoside (X-gal; 80 mg L^-1^). A yeast strain co-transformed with pLexA-53, containing p53, and pB42AD-T, containing SV40 large *T*-antigen, was used as a positive control for protein interaction.

### CyaA Translocation Assay

*eop1, eop3*, and *eop4* genes were amplified by PCR and ligated into the XbaI and SmaI restriction sites of pMJH20 in fusion with the catalytic domain of CyaA from *Bordetella pertussis* (Miao and Miller, 2000). Bacterial strains were transformed with the CyaA fusion constructs, and stable expression was confirmed by immunoblot using anti-CyaA antibody (Santa Cruz Biotechnology; Santa Cruz, CA, United States). Overnight suspensions were adjusted to ≈5 × 10^8^ CFU mL^-1^ in PBS and were syringe-infiltrated into fully expanded leaves of 8-week-old *N. tabacum* cv. Samsun plants. Nine hours after inoculation, leaf disks of 1 cm in diameter were collected from the infiltrated areas, and were immediately frozen in liquid nitrogen. Samples were processed for cAMP quantification as previously described (Schechter et al., 2004). Briefly, frozen leaf disks were ground and resuspended in 250 μL of 0.1M HCl. Suspensions were centrifuged 5 min at 3000 *g* and cAMP levels in supernatants were measured using the cAMP EIA Kit (Cayman Chemical; Ann Arbor, MI, United States) according to the manufacturer’s instructions. Protein concentration in samples was determined using the BCA Protein Assay Kit (Thermo Fisher Scientific; Rockfort, IL, United States). Statistical analyses were done using a one-way analysis of variance, and mean separation was accomplished using the Tukey–Kramer HDS test using JMP 12 (Cary, NC, United States).

### Protein Secretion Assay

Liquid cultures of the WT strain Ea1189 and mutant strains were grown overnight in 50 mL LB medium at 28°C. Cell pellets were resuspended in 40 mL of Hrp-inducing minimal medium (HrpMM), pH 5.7 (Huynh et al., 1989) and induced for 48 h at 24°C. Induced cultures were pelleted and both pellet and supernatants treated with 0.5 mM phenyl methylsulfonyl fluoride (PMSF) and concentrated (300x) using the Amicon Ultra-15 Centrifugal Filter Unit (30 kDa molecular cut-off; Millipore; Billerica, MA, United States). Protein concentrations were measured using the bicinchoninic acid (BCA) Protein Assay Kit. Ten micrograms of proteins in pellet and supernatant were analyzed by SDS-PAGE using a MiniPROTEAN3 system (Bio-Rad, Hercules, CA, United States), and gels were stained with the Pierce^TM^ Silver Stain Kit (Thermo Fisher Scientific; Rockfort, IL, United States).

## Results

### *E. amylovora* TTS Chaperones Interact with Multiple Effector Proteins in Yeast

**FIGURE 1:**
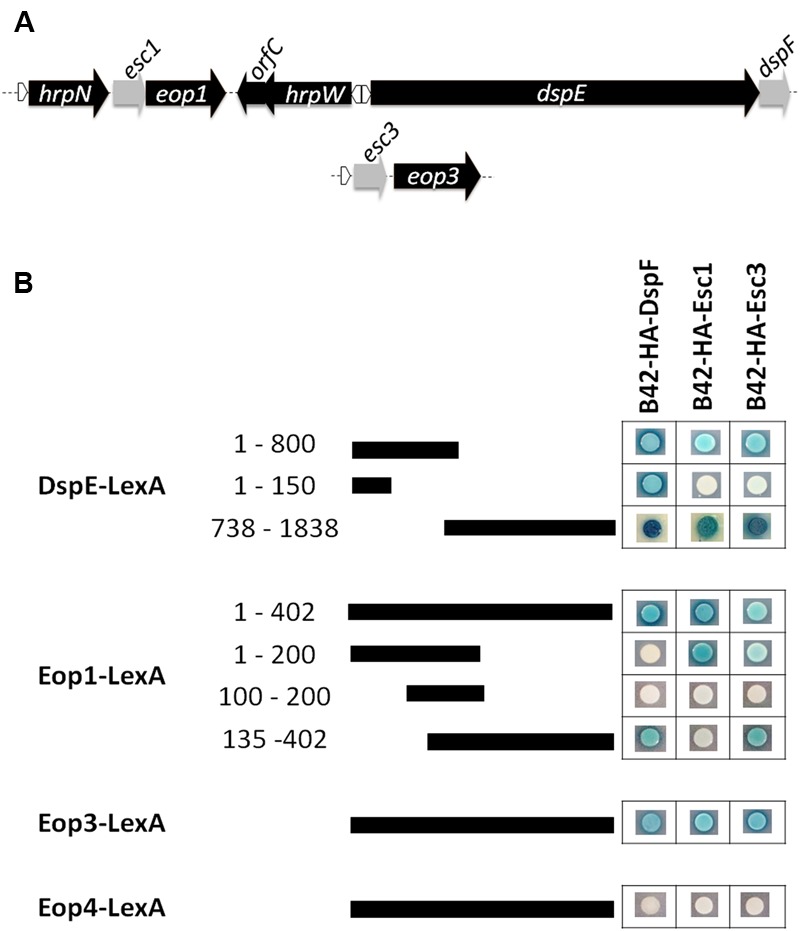
Interactions of TTS chaperones and effector proteins in *E. amylovora*. **(A)** Schematic diagram of gene organization of effector genes *dspE, eop1*, and *eop3* with some adjacent genes and their putative chaperone partners. Depiction of these regions based on analysis of the *E. amylovora* strain ATCC49946 genome (accession number NC_013971.1). Gray-labeled ORFs are confirmed (*dspF*) or predicted to encode putative TTS chaperone proteins (*esc1* and *esc3*). White triangles indicate the presence of a *hrpL-*regulated promoter. **(B)** Yeast two-hybrid interactions between prey fusions of DspF, the putative chaperones Esc1 and Esc3 in the pB42AD vector, and bait fusions of the effector proteins Eop1, Eop3, Eop4, N- terminal portions of DspE in the pGilda vector. Pairs of prey and bait fusions were transformed in the EGY48 yeast strain and selected on SD-galactose/raffinose medium amended with -Ura/-His/-Trp/-Leu dropout and Xgal (80 mg L^-1^). Positive interactions were identified when a yeast colony harboring a particular prey/bait fusion pair turned blue.

TTS chaperone genes have often been found as short open reading frames (ORFs) located adjacent to the genes encoding their cognate effector proteins (Frithz-Lindsten et al., 1995; Cornelis, 2006). Kim and Beer (1998) reported the presence of two putative TTS chaperone genes named *orfA* and *orfC* located adjacent to *eop1* and the harpin gene *hrpW*, respectively (**Figure 1A**). Similarly, Nissinen et al. (2007) reported the presence of a gene downstream of a *hrpL*-regulated promoter and located within an operon with the effector gene *eop3*, which shares homology with the TTS chaperone gene *shcF* from *P. syringae* pv. *tomato* (Shan et al., 2004) (**Figure 1A**). Further analyses of this putative TTS chaperone gene including prediction of the secondary structure for this 137-amino acid chaperone protein using the software 3D-Jury (Ginalski et al., 2003) and the Phyre server (Kelley and Sternberg, 2009) indicated the presence of three α-helical motifs and an acidic pI, both characteristic of TTS chaperones supporting a role as secretion chaperone for the protein encoded by this gene. Conversely, the secondary structure predicted for the protein encoded by *orfC* did not match the structural characteristics of TTS chaperones. In addition, sequence annotation and secondary structure analyses of genes surrounding the secreted effector Eop4 and the putative effector gene *hopPtoC_Ea_* (Zhao et al., 2005) did not reveal the presence of any ORF with the characteristics of a TTS chaperone gene. These results indicate that in addition to DspE, two other effector proteins in *E. amylovora* are encoded adjacent to confirmed or putative chaperone genes. Because these effector proteins are named Eop1 and Eop3, we propose the putative genes encoding chaperone proteins be named *esc1* and *esc3* for *Erwinia* secretion chaperones 1 and 3, respectively.

Similar to other TTS chaperone proteins, DspF has been shown to interact with more than one effector protein in yeast two-hybrid experiments (Asselin et al., 2006). In order to assess whether DspF, Esc1, and Esc3 interact with multiple TTS effector proteins in *E. amylovora*, we performed a series of yeast two hybrid analyses. All of the evaluated chaperone proteins fused with a B42-hemagglutinin (HA) tag interacted with fusions of the N-terminal portion of DspE with the LexA binding domain [DspE_(1-800)_-LexA], the C-terminal portion of DspE (DspE_(738-1838)_-LexA), Eop1-LexA, and Eop3-LexA, but did not interact with Eop4-LexA (**Figure 1B**). In contrast with DspF, which interacts with residues 51- 100 of DspE as previously reported (Triplett et al., 2009; Oh et al., 2010), B42-HA-Esc1 and B42-HA-Esc3 did not interact with the DspF-binding domain in the N terminal region of DspE-LexA (**Figure 1B**), indicating that the interaction domain for these chaperones is not shared with DspF and is located elsewhere in the effector. Indeed, a strong interaction of DspE_(738-1838)_-LexA with B42-HA-Esc1was detected, in agreement with similar results observed by Oh and collaborators with a DspE_780-1838_-LexA fusion (Oh et al., 2010), and with B42-HA-Esc3 as well. Interestingly, an interaction of DspF with the C-terminal portion of DspE (residues 738–1838) was detected, suggesting that this chaperone protein has multiple binding regions along the effector protein. The chaperone binding domains (CBD) of the Eop1 effector were mapped with further yeast studies. While the N-terminal 200 residues of Eop1 interacted strongly with its partner chaperone B42-HA-Esc1, no interaction with B42-HA-DspF and B42-HA-Esc3 was observed. Conversely, interaction of residues 135 – 402 in the C terminus of Eop1 with B42-HA-DspF and B42-HA-Esc3 was evidenced, while no interaction with B42-HA-Esc1 was observed (**Figure 1B**).

### Simultaneous Expression of *dspF, esc1*, and *esc3* Genes Is Required for Full Secretion of DspE and Translocation of a DspE_(1-737)_-CyaA Reporter

We previously used an adenylate cyclase reporter (CyaA) to demonstrate that the N-terminal CBD of DspE, is stably expressed and translocated into tobacco cells by the WT strain *E. amylovora* Ea1189 (Triplett et al., 2009). To assess the importance of DspF, Esc1, and Esc3 for the successful delivery of DspE into host cells, we compared the secretome of the WT Ea1189 and the different mutant strains when grown under *hrpL-*inducing conditions, and the translocation levels of the DspE_(1-737)_-CyaA fusion from the same strains to tobacco plants, as described in the methods section.

**FIGURE 2:**
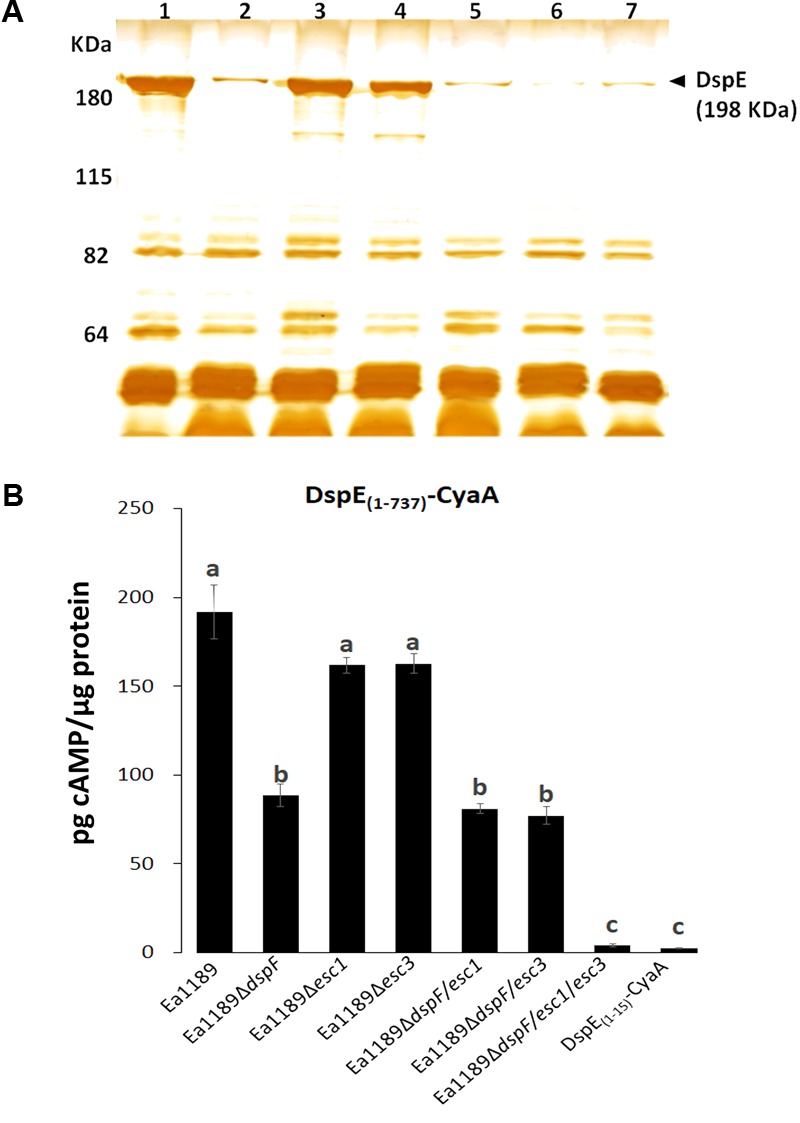
Effect of TTS chaperones in secretion of DspE and translocation of DspE_(1-737)_-CyaA. **(A)** Supernatant protein profiles of Ea1189 (1), Δ*dspF* (2), Δ*esc1* (3), Δ*esc3* (4), Δ*dspF/esc1* (5), Δ*dspF/esc3* (6), and Δ*dspF/esc1/esc3* (7) after 48 h of growth in HrpMM, to induce the expression of the TTSS. Supernatant fractions were separated from pellets by centrifugation and equal amounts of protein (10 micrograms) were analyzed by SDS-PAGE. The assay was repeated twice with similar results. **(B)** cAMP accumulation in tobacco leaves inoculated with Ea1189, and mutant strains expressing DspE_(1-737)_-CyaA at 9 hpi. Ea1189 expressing DspE_(1-15)_-CyaA was used as negative control. Leaf samples were collected using a 1 cm diameter core borer and immediately frozen in liquid nitrogen for posterior processing as described in Section “Materials and Methods.” Results are the means and error bars represent the SED. Different letters above bars denote statistically significant differences (Tukey–Kramer HDS test, *P* < 0.05). The assay was done twice with similar results.

A reduction in DspE intracellular accumulation in the absence of DspF has been previously reported (Gaudriault et al., 2002). Moreover, secretion profiling revealed that, although DspE was secreted by all the strains tested in this study, seen by the presence of a previously characterized unique 198 kDa band (Gaudriault et al., 2002; Nissinen et al., 2007), secretion of this effector was apparently reduced in the double mutants Ea1189Δ*dspF/esc1* and Ea1189Δ*dspF/esc3*, and in the triple chaperone gene mutant Ea1189Δ*dspF/esc1/esc3*, when compared with the single Ea1189Δ*dspF* mutant (**Figure 2A**). Secretion of DspE was not impaired in single mutants Ea1189Δ*esc1* and Ea1189Δ*esc3* when compared with the WT strain. Furthermore, while cAMP accumulation due to translocation of DspE_(1-737)_-CyaA from the Δ*esc1* and Δ*esc3* single mutants was not significantly different from the Ea1189 WT, significantly reduced levels of cAMP were observed for Ea1189Δ*dspF* and for both double mutants deleted in *dspF* and *esc1* or *esc3* (**Figure 2B**). The lowest level of translocation observed was for the Ea1189Δ*dspF/esc1/esc3* triple mutant which was not significantly different from the DspE_(1-15)_-CyaA fusion, previously reported to be a non-translocated fragment of DspE (Triplett et al., 2009). Overall, these results indicate that TTS chaperones in *E. amylovora* act additively in order to effectively secrete DspE to the extracellular milieu and to translocate this effector protein into the host cytoplasm through the TTSS.

### Esc1 and Esc3 Do Not Affect *E. amylovora* Pathogenicity

**FIGURE 3:**
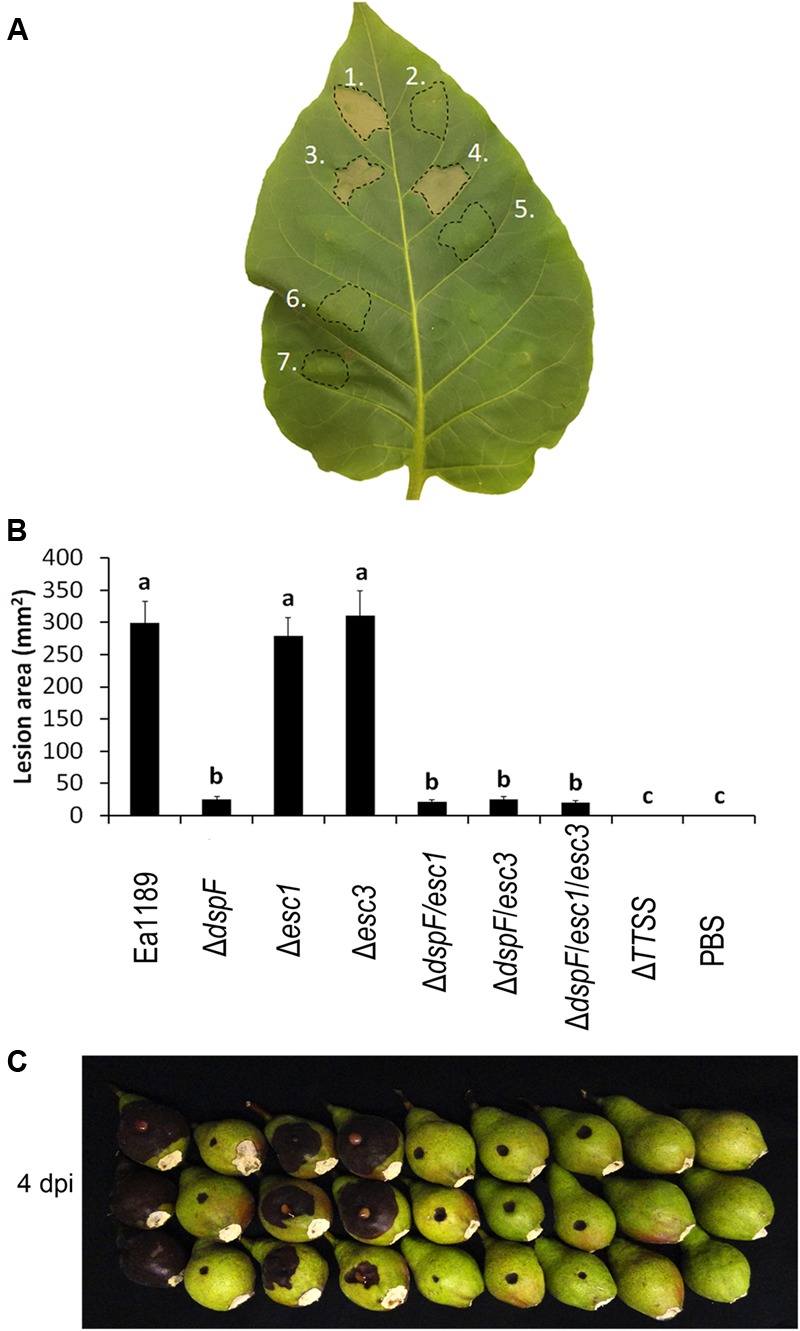
Effect of the TTS chaperones DspF, Esc1 and Esc3 on the virulence in E. amylovora. **(A)** Tobacco leaf cell death 24 h after infiltration with Ea1189 (1), Ea1189Δ*dspF* (2), Ea1189Δ*esc1* (3), Ea1189Δ*esc3* (4), Ea1189Δ*dspF/esc1* (5), Ea1189Δ*dspF/esc3* (6), and Ea1189Δ*dspF/esc1/esc3* (7). **(B)** Lesion size on immature pears inoculated with 3 μL of the same strains at ≈1 × 10^4^ CFU mL^-1^. Ea1189ΔTTSS strain was used as negative control. Lesions were photographed and the area was quantified at 4 dpi using ImageJ software. The experiment was repeated three times with similar results. Results are the means and error bars represent the SED. Different letters above bars denote statistically significant differences (Tukey–Kramer HDS test, *P* < 0.05). **(C)** Symptom development on stab-wounded immature pears at 4 dpi with Ea1189 and mutant strains.

The pathogenicity of *E. amylovora* has been reported to be dramatically reduced in a *dspF* deletion mutant when compared with the WT strain (Gaudriault et al., 2002; Triplett et al., 2009). However, a small amount of the N-terminal portion of DspE is translocated in the absence of this chaperone protein, and a deletion mutant of *dspF*, although less aggressive than the WT, is still pathogenic (Triplett et al., 2009). To determine whether the additional TTS chaperone proteins Esc1 and Esc3 have an additive effect in the efficient translocation of DspE and hence, an additive impact on the pathogenicity phenotype of *E. amylovora*, a series of mutant strains was constructed and evaluated in an immature pear disease model and for induction of hypersensitive-like cell death in tobacco leaves. While inoculation with the Ea1189 WT, Ea1189Δ*esc1*, and Ea1189Δ*esc3* resulted in severe tobacco leaf collapse at 24 hpi, inoculation with Ea1189Δ*dspF* and all Ea1189Δ*dspF-*derived double and triple chaperone mutants triggered no signs of necrosis (**Figure 3A**). Similarly, single deletions of *esc1* and *esc3* did not have a significant effect on pathogenicity on immature pears, whereas double deletion mutants Ea1189Δ*dspF/esc1* and Ea1189Δ*dspF/esc3* showed a reduction of aggressiveness that was statistically equivalent with the reduction in aggressiveness in the *dspF* mutant background (**Figures 3B,C**). Interestingly, a mutant strain lacking the three TTS chaperone genes still caused disease at the same level as double deletion mutants.

### DspF Negatively Affects the Translocation of Eop1-CyaA and Eop3-CyaA, But Not Eop4-CyaA

**FIGURE 4:**
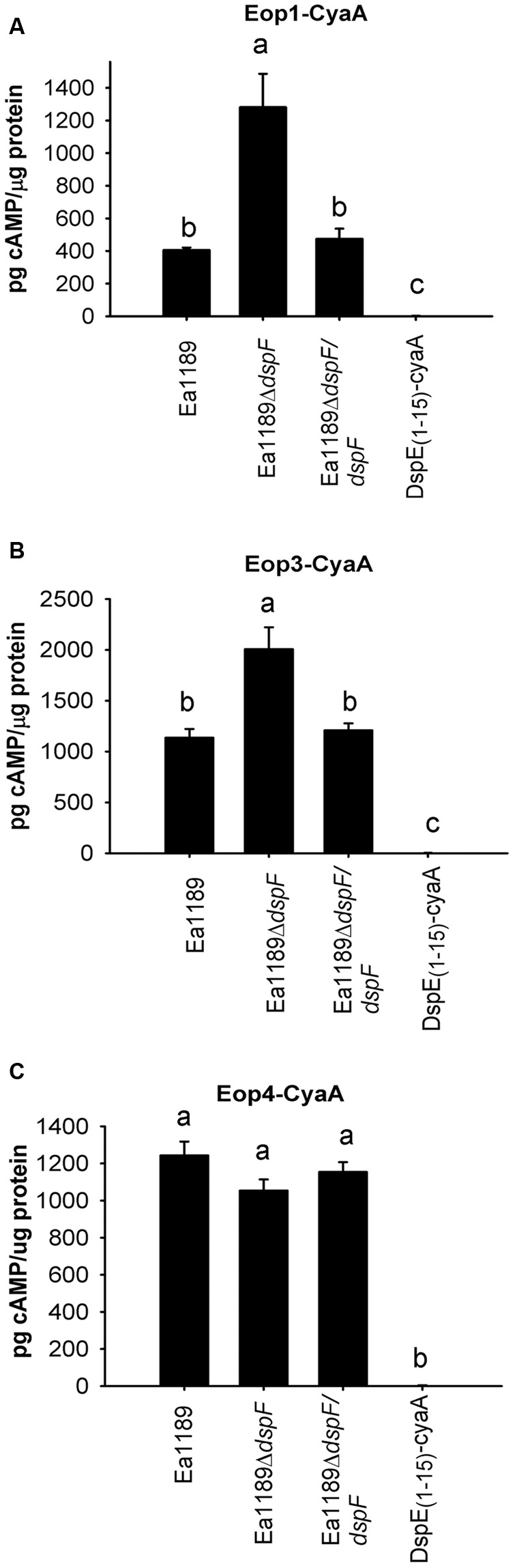
Effect of the TTS chaperones DspF on the traslocation of Eop1-CyaA, Eop3 -CyaA and Eop4-CyaA fusion proteins. cAMP accumulation was measured at 9 hpi on tobacco leaves infiltrated with Ea1189, Ea1189Δ*dspF* and Ea1189Δ*dspF/dspF*, expressing Eop1-CyaA **(A)**, Eop3-CyaA **(B)** and Eop4-CyaA **(C)**. Ea1189 expressing DspE_(1-15)_-CyaA was used as negative control. Leaf samples were collected using a 1 cm diameter core borer and immediately frozen in liquid nitrogen for posterior processing Results represent the means and error bars represent the SED. Different letters above bars denote statistically significant differences (Tukey–Kramer HDS test, *P* < 0.05). The experiment was done twice with similar results.

To determine whether DspF affects the translocation of the effector proteins Eop1, Eop3 and Eop4, accumulation of cAMP in tobacco leaf fusions was evaluated after infiltration of Ea1189Δ*dspF* containing either Eop1-CyaA, Eop3-CyaA, or Eop4-CyaA. Surprisingly, translocation of Eop1-CyaA and Eop3-CyaA was significantly increased from Ea1189Δ*dspF* compared to the WT Ea1189 or to the Ea1189Δ*dspF*/*dspF* complemented strain (**Figures 4A,B**). On the other hand, cAMP accumulation due to Eop4-CyaA translocation was not affected by the deletion of *dspF* (**Figure 4C**). These observations suggest that DspF might play a regulatory role in the translocation of Eop1 and Eop3.

## Discussion

The TTSS exhibits various mechanisms of regulation at every stage in the assembly and translocation processes (Cornelis, 2006). In fact, a hierarchical organization in effector protein delivery through the TTSS has been demonstrated for several animal pathogens (Mills et al., 2008; Lara-Tejero et al., 2011; Portaliou et al., 2017). However, mechanisms regulating TTSS assembly and the translocation of pre-formed proteins in plant pathogenic bacteria are still poorly understood. In this study, we examined the roles of DspF and two other TTS chaperones, Esc1 and Esc3, for interactions with effector proteins, effects on secretion and translocation of effectors, and effects on bacterial pathogenicity.

Although many TTS chaperones interact with a single effector protein (class IA), class IB TTS chaperones that bind to multiple target effectors (multiple cargo) have been described in several animal pathogenic bacteria. Examples of chaperones that can bind multiple effectors include SrcA and InvB from *Salmonella enterica* serovar Typhimurium and CesT from enteropathogenic *Escherichia coli* (Bronstein et al., 2000; Creasey et al., 2003; Ehrbar et al., 2004; Thomas et al., 2005; Cooper et al., 2010). Plant pathogen examples include HpaB from *X. campestris* pv. *vesicatoria*, and ShcS1 and ShcO1 from *P. syringae* pv. *tomato* (Büttner et al., 2004; Kabisch et al., 2005; Büttner et al., 2006). Our yeast two-hybrid studies suggest that DspF, Esc1, and Esc3 belong to the class IB TTS chaperone category, as they bind not only to their cognate effector partner, but also seem to be functioning as multi-cargo chaperones. In the case of DspE, these TTS chaperones function cooperatively in DspE cellular trafficking and translocation into the plant cell. This finding is consistent with previous studies in *Chlamydia pneumoniae* showing that the TTS chaperones Ssc1 and Ssc4 bind forming a complex that interacts with the N-terminal region of the effector protein CopN, promoting CopN secretion through the TTSS (Silva-Herzog et al., 2011). Similarly, the TTS chaperones EscH and EscS from *Edwardsiella piscicida* have be demonstrated to interact with the effector protein EseK, enhancing secretion and translocation into host cells (Cao et al., 2017).

In a previous report, we mapped a CBD for DspF to residues 51- 100 in the N terminus of DspE (Triplett et al., 2009). Interestingly, yeast two-hybrid results suggest that, in addition to the N terminal-localized CBS, DspF interacts with at least one additional domain of DspE. Since one the main roles of TTS chaperones is the stabilization of the cognate effector within the bacterial cytoplasm, it is not surprising that DspF might bind to multiple regions along the length of DspE, especially given the large size of this effector protein (1838 residues). Moreover, our results suggest that the CBDs for Esc1 and Esc3 are not located in the N-terminal portion of DspE, but are located elsewhere in the effector protein, ruling out the possibility of heterodimerization with DspF for binding in this specific location of the effector. The presence of CBDs in non-N-terminal effector regions has been reported previously including in *P. syringae* pv. *tomato* for the TTS chaperones ShcO1, ShcS1, and ShcS2, which bind to the middle third portion of HopO1-1 (Guo et al., 2005), and for CT548, a TTS chaperone from *Chlamydia trachomatis*, that binds to the central region of CT082, a type III substrate (Pais et al., 2013).

Echoing the specificity of DspE N-terminal CBD for the cognate chaperone DspF, the CBD in residues 1- 100 of the effector Eop1 were only bound by the cognate chaperone Esc1, while DspF and Esc3 binding sites are likely located within the last 200 residues of this effector. Although it has been previously reported that DspF is indispensable for stable expression of DspE in *E. amylovora* cells and for secretion to the extracellular milieu, as this effector protein was not detected by immunoblot analyses in whole cell lysates or culture supernatants of a *dspF* mutant strain (Gaudriault et al., 2002), our studies indicated that the full-length DspE can be expressed and secreted in the absence of DspF, at lower levels than the WT strain (**Figure 3A**). This discrepancy can be explained by the differences between the approaches used to detect the protein and their detection thresholds. Moreover, the fact that a *dpsF* mutant strain retains some pathogenicity while a *dspE* mutant does not (Gaudriault et al., 2002; Triplett et al., 2009), supports our observation that DspE can be expressed, secreted, and translocated in a DspF-independent fashion.

The capacity of the N-terminal region of DspE for DspF-independent translocation previously observed (Triplett et al., 2009), and the interaction of LexA-DspE_(1-800)_ and LexA-DspE_(738-1838)_ with B42-HA-Esc1 and B42-HA-Esc3 observed in this study, led us to hypothesize that TTS chaperone proteins other than DspF might also be involved in the efficient translocation of DspE into the host cell. While deletions of *esc1* or *esc3* do not have a significant effect on pathogenicity, our secretion and translocation assays indicated that the activity of the TTS chaperones on DspE secretion and translocation is additive, as secretion of DspE was visibly diminished from the double mutants Ea1189Δ*dspF/esc1* and Ea1189Δ*dspF/esc3* and the Δ*dspF/esc1/esc3* triple mutant, and the Δ*dspF/esc1/esc3* triple mutant strain permits less translocation of DspE_(1-737)_-CyaA translocation than single or double chaperone mutants. It should be noted that for all of our translocation studies we used an N-terminal portion of DspE rather than the full-length protein, and that the translocation efficiency of the N-terminal reporter could differ from that of the intact protein. Our results present primary evidence of TTS chaperone cooperative behavior for the translocation of DspE, and further studies with the full-length effector would complement these findings.

In contrast to DspE_(1-737)_-CyaA and Eop4-CyaA, our experiments indicated that translocation of Eop1-CyaA and Eop3-CyaA is negatively affected by DspF. These results suggest that DspF might play an antagonistic role, delaying the translocation of effectors other than DspE, and establishing a hierarchy for effector export. In a recent study, Portaliou et al. (2017) demonstrated that the TTS chaperone association of SepD with the effector protein SepL in enteropathogenic *E. coli* is critical for the temporal regulation of TTS substrate passage through the translocase channel. Furthermore, the multi-cargo chaperone HpaB in *X. campestris* pv. *vesicatoria* has been determined to function as a regulator of the recognition of translocation signals independently of its TTS chaperone role (Scheibner et al., 2017). The mechanism of DspF-dependent regulation of translocation remains unknown, and further studies would be helpful in determining if this regulation involves differences in chaperone-effector affinities or regulation at the transcriptional, translational or post-translational levels. In addition, several studies have postulated Eop1 and Eop3 as effector proteins exhibiting avirulence functions (Asselin et al., 2011; Bocsanczy et al., 2012) which might explain the antagonistic role of DspF on these effector proteins.

In this study we took advantage of the small number of TTS effector and chaperone proteins produced by *E. amylovora* in order to investigate the interactions that mediate effector cellular trafficking and extracellular export and their implications in bacterial pathogenicity. We determined that the TTS chaperones DspF, Esc1 and Esc3 exhibit features of multi-cargo and that cooperation exists between them in order to efficiently deliver the TTS effector DspE into plant cells by *E. amylovora*. Moreover, our findings suggest that in addition to enhancing DspE delivery to the host cell through the TTSS, DspF exerts additional regulatory roles on other effectors proteins, delaying their translocation and thus modulating the timing of effector export. Further studies are needed to determine how *E. amylovora* orchestrates hierarchical secretion and translocation of effectors to colonize its host and cause disease.

## Author Contributions

LC, LT, and GS conceived and designed the experiments. LC and LT performed the experiments and analyzed the data. LC, LT, and GS wrote the manuscript.

## Conflict of Interest Statement

The authors declare that the research was conducted in the absence of any commercial or financial relationships that could be construed as a potential conflict of interest.
